# Delving into the Role of lncRNAs in Papillary Thyroid Cancer: Upregulation of LINC00887 Promotes Cell Proliferation, Growth and Invasion

**DOI:** 10.3390/ijms25031587

**Published:** 2024-01-27

**Authors:** Cristina Tous, Carmen Muñoz-Redondo, Angela Gavilán, Nereida Bravo-Gil, Fátima Baco-Antón, Elena Navarro-González, Guillermo Antiñolo, Salud Borrego

**Affiliations:** 1 Department of Maternofetal Medicine, Genetics and Reproduction, Institute of Biomedicine of Seville, University Hospital Virgen del Rocío/CSIC/University of Seville, 41013 Seville, Spain; 2 Center for Biomedical Network Research on Rare Diseases (CIBERER), 41013 Seville, Spain; 3 Department of Endocrinology and Nutrition, University Hospital Virgen del Rocío, 41013 Seville, Spain

**Keywords:** lncRNA, papillary thyroid carcinoma, CRISPR-CAS9, linc00887, PD-L1, cancer

## Abstract

Papillary thyroid carcinoma (PTC) is the most common histological category of thyroid cancer. In recent years, there has been an increasing number of studies on lncRNAs in PTC. Long intergenic non-protein coding RNA 887 (LINC00887) is a critical oncogene in developing other cancers. LINC00887 is upregulated in PTC samples but its role in PTC is currently unclear. This study aimed to investigate the impact the disruption of LINC00887 expression has on PTC progression. We performed a CRISPR/Cas9 strategy for the truncation of LINC00887 in BCPAP and TPC1 cell lines. Functional assays showed that LINC00887 knockdown in both TPC1 and BCPAP cells reduced cell proliferation, colony formation and migration, delayed the cell cycle, and increased apoptosis. These results strengthened the role of LINC00887 in cancer and showed for the first time that this lncRNA could be a potential oncogene in PTC, acting as a tumor promoter. Modulation of the immune system may be one of the etiopathogenic mechanisms of LINC00887 in PTC, as shown by the observed influence of this lncRNA on PD-L1 expression. In addition, the biological pathways of LINC00887 identified to date, such as EMT, the Wnt/β-catenin signaling pathway or the FRMD6-Hippo signaling pathway may also be relevant regulatory mechanisms operating in PTC.

## 1. Introduction

Thyroid cancer (TC) is the most common endocrine malignancy (90% of all endocrine neoplasms) [1,2]. Compared to other cancers, TC can be considered a rare tumor with a range of 2–20 cases per 100,000 persons per year [3]. According to histopathological classification, papillary thyroid carcinoma (PTC) is a differentiated thyroid cancer that originates from the follicular cell epithelium of the thyroid [3,4] and is the most common category of TC, accounting for 80–85% of all cases [2]. PTC has a low degree of malignancy; in fact, the five-year survival rate of PTC is approximately 99.5%, but recurrence and distant metastases increase the death risk [4,5]. PTC often metastasizes to regional lymph nodes, and distant metastases are rare. However, when it does occur, the prognosis of PTC becomes poor and the survival rate can be reduced to 74% (according to https://www.cancer.org, accessed on 1 June 2023). The main sites of distant metastasis of PTC are usually the lungs and bones [6]. Conventional treatment options for PTC patients include surgery, chemotherapy and radioactive iodine therapy, however, these strategies are currently ineffective in patients with metastatic PTC [7]. Therefore, the investigation of novel biomarkers in PTC is of great importance.

Recently, there has been particular interest in the regulation of TC pathogenesis by long non-coding RNAs (lncRNAs) and the resulting clinical implications of their biological functions [8,9]. LncRNAs refer to a class of non-coding RNA transcripts greater than 200 nucleotides in length. Although they are considered to be incapable of encoding proteins, they are currently in the spotlight due to their recently acquired roles as crucial players in the transcriptional, epigenetic, or post-transcriptional regulation of gene expression [10]. Indeed, they regulate gene expression at different levels and by different mechanisms, affecting apoptosis, cell cycle, cell proliferation, and differentiation, participating in both biological and pathological processes, and spanning tumor-associated pathways [11,12]. Alterations within lncRNAs exhibit tumor suppressor or carcinogenic effects and play an important role in the development of tumor [13]. Therefore, lncRNAs are promising and powerful targets for cancer prognosis and treatment [14].

There has been an increasing number of studies of lncRNAs in PTC since 2007, although most of them have emerged in the last few years. For example, LINC00163, LINC01638 and RNA HOX transcript antisense RNA (HOTAIR) were upregulated in PTC and their knockdown suppressed the cell proliferation, invasion and migration by modulating epithelial-mesenchymal transition (EMT) and/or Wnt/β-catenin signaling pathway [15,16,17,18]. Specifically, the implication of lncRNA in PTC has been previously investigated in our group, to which the LncRNA lung cancer-associated transcript 1 (LUCAT1) was found to be a novel prognostic biomarker that was upregulated in PTC patients [3]. Among the lncRNAs highlighted in this screening, we also found the LncRNA long intergenic non-protein coding RNA 887 (LINC00887). This LncRNA was dramatically upregulated in PTC tissues compared with normal tissues [3].

LINC00887, also known as linc-ATP13A4-8 and HEIRCC [19], is located on the antisense strand of chromosome 3 and consists of 4 exons and 3 introns (ENST00000414120.1). To date LINC00887 has been associated with various tumors and has been described to be upregulated in renal cell carcinoma (RCC) [20,21,22], nasopharyngeal carcinoma [23], glioma [24], lung carcinoma [25] and tongue squamous cell carcinoma [26]. In contrast, LINC00887 was downregulated in cervical cancer [27]. In all these cases, the alteration of LINC00887 expression significantly affected the proliferation, migration and invasion of these carcinomas and is a candidate for prognostic value in cancer.

Due to their aberrant expression in cancers, long non-coding RNAs (lncRNAs) are crucial for tumor immunotherapy since they modulate the immune system and increase resistance to immunotherapy [28]. Other immune regulators may also be altered in cancer, such as PD-L1. Programmed death-ligand 1 (PD-L1) is a transmembrane protein which plays a crucial role in inhibiting adaptive immune responses [28]. As PD-L1 is an immune checkpoint molecule, it can be used by cancer cells to thwart the anti-tumor actions of the immune system. Wu J. et al. proposed that LINC00887 was upregulated in clear cell renal cell carcinoma (ccRCC), and that knockdown of LINC00887 resulted in decreased PD-L1 expression, increased CD8 T cell toxicity, decreased levels of apoptosis, and enhanced chemotaxis. Furthermore, these authors also found that LINC00887 inhibited immune infiltration of CD8 cells in clinical tissues [20]. On the other hand, PD-L1 overexpression was observed in PTC patients and was significantly associated with aggressive clinicopathological parameters [28].

In this study, we aimed to elucidate the role of LINC00887 in PTC and to clarify its molecular mechanism in the development of PTC. To this end, we performed a series of in vitro assays to investigate how LINC00887 knockdown affected cell proliferation, invasion, cell cycle progression, and PD-L1 expression. This study is the first to investigate the functional role of LINC00887 in PTC and provides new insights into the pathogenesis of this carcinoma [14].

## 2. Results

### 2.1. Isoform Detection and In Vivo Truncation of LINC00887 via Genome Editing

**Figure 1:**
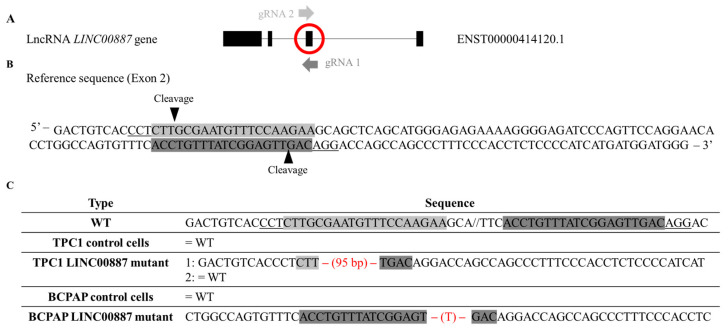
Schematic representation of the expressed LINC00887 isoform, CRISPR/Cas9 design, targets and selected mutant clones. (**A**) Schematic representation of the LINC00887 isoform expressed in TPC1 and BCPAP cell lines and gRNA targets for CRISPR/Cas9-mediated mutagenesis of this gene. Exons and introns are indicated by black boxes and lines, respectively. (**B**) DNA sequence region of exon 2 LINC00887 gene. Two CRISPR/Cas9 target sequences (gRNA 1 and gRNA 2) are shaded in two different shades of grey. The protospacer adjacent motif (PAM: 5′-NGG-3′) sequences are underlined. Black arrows indicate putative cleavage sites of the gRNAs. (**C**) The DNA sequence of the target regions for each cell line clone. Mutations are indicated in red. Hyphens indicate a deletion. Base pairs (bp) in brackets indicate the length of the deletion.

We found 11 different isoforms for LINC00887 according to Ensembl (https://www.ensembl.org/index.html; accessed on 20 September 2022), each with a currently undescribed function. Therefore, the first step was to determine the isoform of LINC00887 expressed in TPC1 and BCPAP cell lines by performing Sanger sequencing using several pairs of primers (Supplementary Table S1). These primers spanned each exon, revealing the presence of four exons corresponding to transcript ID ENST00000414120.1 according to Ensembl https://www.ensembl.org/index.html; accessed on 20 September 2022 (Figure 1A).

According to the LINC00887 gene structure (Figure 1A), gene editing by CRISPR/Cas9 was performed using two guides against exon 2 (Figure 1B). We selected two clones that were genotyped by Sanger sequencing and their changes in the exon 2 sequence are shown in Figure 1C. DNA analysis of targeted regions in two clones revealed two different mutagenesis patterns. BCPAP LINC00887 mutant showed a single base (T) pair deletion in homozygosis, whereas TPC1 LINC00887 mutant showed a 95 bp deletion in heterozygosis.

### 2.2. Expression of LINC00887 in Papillary Thyroid Cancer

**Figure 2:**
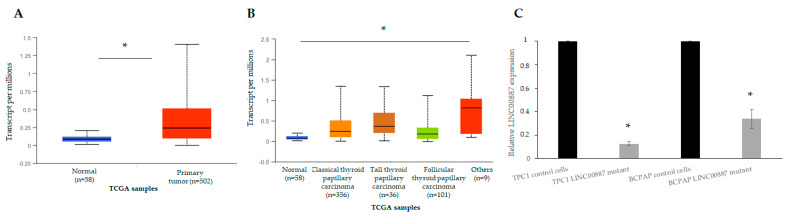
Expression of LINC00887 in thyroid cancer compared to normal thyroid tissue. Comparison of the expression of LINC00887 in thyroid cancer versus normal thyroid tissue (**A**) and different thyroid subtypes (**B**) using the UALCAN database with TCGA data. (**C**) Expression of LINC00887 in control cells as well as in CRIPR/Cas9 edited cells. Data are presented as the mean ± SD based on more than three independent experiments (* *p* < 0.05).

The qRT-PCR assay was performed to evaluate the LINC00887 expression. In an expression array performed in our group between normal and tumor tissue of patients with PTC [3], we observed that LINC00887, like other lncRNA, was overexpressed. Therefore, we evaluated the expression pattern of LINC00887 in normal tissue versus TC, as well as among different TC subtypes (PTC, FTC and others), using the UALCAN website. The results showed that LINC00887 levels were higher in TC tissue compared to normal samples (Figure 2A) and had a tendency to be increased in all TC subtypes, with greater expression in classical and tall PTC (Figure 2B).

Analysis of LINC00887 expression revealed that the BCPAP cell line presented a higher expression level than the TPC1 cell lines. However, depletion of the LINC00887 gene was performed in both lines and a significant decrease in mRNA was observed in both mutants compared to their respective control cells (Figure 2C, * *p* < 0.05).

### 2.3. The Role of LINC00887 in Cell Proliferation and Colony Formation

**Figure 3:**
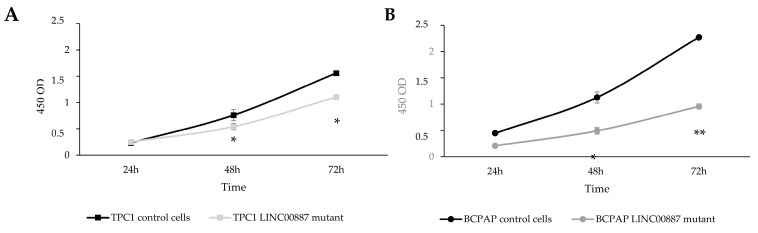
Effect of LINC00887 downregulation on cell proliferation. Proliferation was assessed by CCK-8 assay in TPC1 (**A**) and BCPAP (**B**) cells. Data are presented as the mean ± SD based on more than three independent experiments (* *p* < 0.05, ** *p* < 0.01).

CCK-8 assay was performed to assess the proliferative capacity of LINC00887 mutant cells. As shown in Figure 3, LINC00887 knockdown significantly decreased proliferation in both cell lines compared to the control group (Figure 3, * *p* < 0.05 and ** *p* < 0.01).

**Figure 4:**
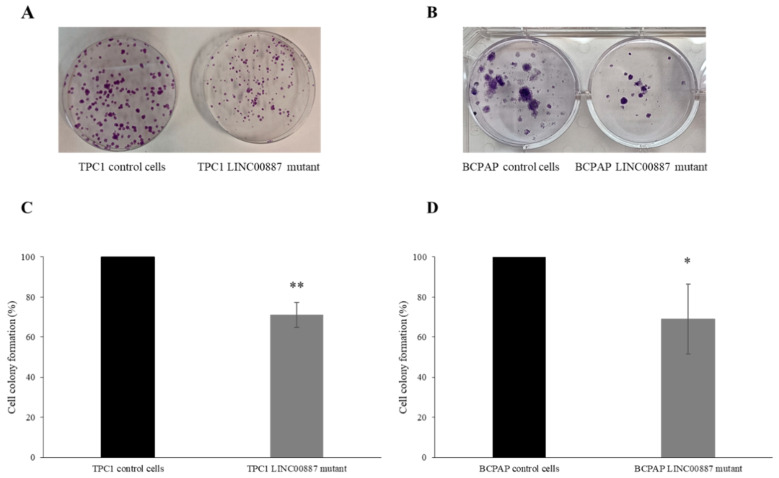
LINC00887 knockdown impacts on TPC1 and BCPAP cell colony formation. Representative images are shown for colony formation assays of TPC1 (**A**) and BCPAP (**B**) cells. Analysis comparison of colony numbers in TPC1 (**C**) and BCPAP (**D**) cells. Data are presented as the mean ± SD based on more than three independent experiments (* *p* < 0.05, ** *p* < 0.01).

In addition, a colony formation assay was performed to determine the ability of the cells to form colonies. LINC00887 mutant cells reduced the number of cell colonies in both cell lines. Interestingly, the colonies of BCPAP cells were found to be bigger than those of TPC1 cells, and in both TPC1 and BCPAP LINC00887 mutant cells, the colony size was smaller compared to control conditions. In addition, LINC00887 mutant colony formation was significantly reduced compared to the mock group (Figure 4, * *p* < 0.05, ** *p* < 0.01).

### 2.4. LINC00887 Knockdown Disrupted Cell Migration and Invasion

**Figure 5:**
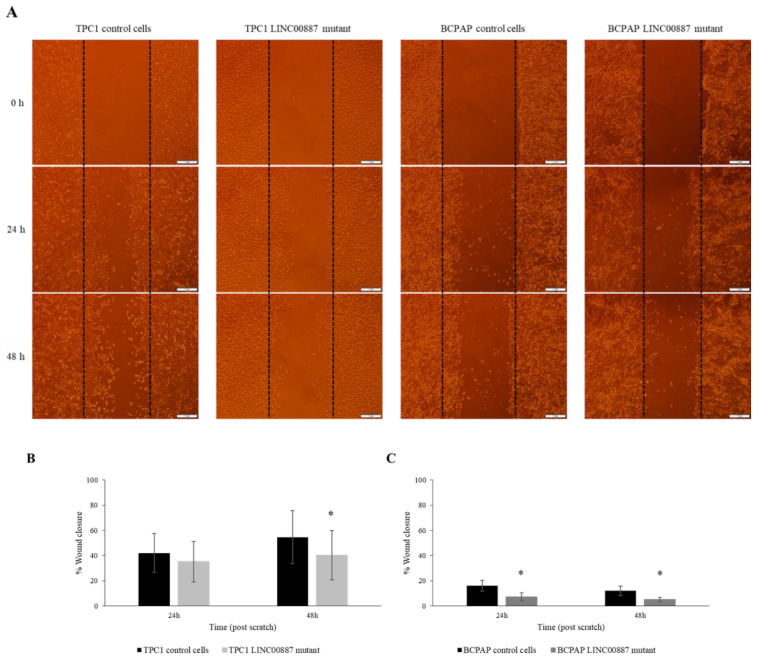
The downregulation of LINC00887 inhibits the migration of cancer cells. (**A**) Images of the scratched area at different time points (time 0 h, 24 h and 48 h). Dotted lines indicate the initial wound area. Original magnification: ×4. Scale bar = 2 mm. Semi-quantitative analysis of wound closure by measuring the width of the wounds both in TPC1 (**B**) and BCPAP (**C**) cells. Data are presented as the mean ± SD based on more than three independent experiments (* *p* < 0.05).

The invasion ability of TPC1 and BCPAP cells was assessed using a wound-healing assay. Quantitative analysis of wound-healing results showed 20% less invasion than control cells, respectively, indicating that the downregulation of the LINC00887 gene resulted in less wound closure and, therefore, less growth and invasion of the tumorigenic cells. (Figure 5, * *p* < 0.05).

### 2.5. Effect on Cell Cycle and Apoptosis by LINC00887

**Figure 6:**
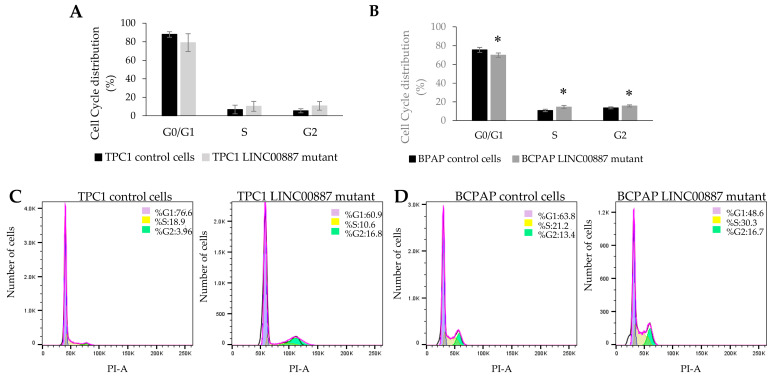
Downregulated LINC00887-induced cell cycle arrest. Graphical representation of cell cycle distribution for the cell populations of TPC1 (**A**) and BCPAP (**B**) cells. Cell cycle profile for TPC1 (**C**) and BCPAP (**D**) cells, in which each population is represented with a different color (G1-phase: purple; S-phase: yellow and G2-phase: green). Data are presented as the mean ± SD based on more than three independent experiments (* *p* < 0.05).

**Figure 7:**
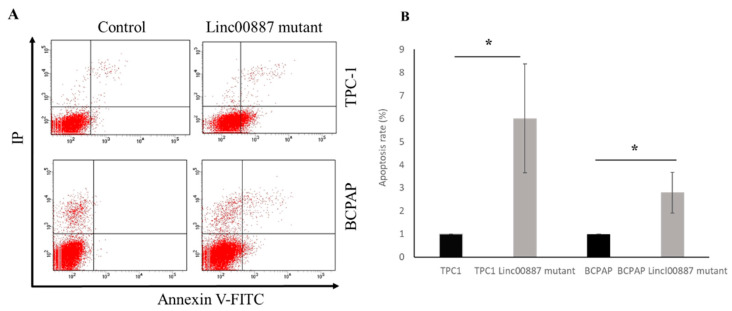
Effects on TPC-1 and BCPAP apoptosis of LINC00887 knockdown. (**A**) Flow cytometric analyses of cell apoptosis using Annexin V-FITC and propidium iodide both for controls and mutants of TPC1 and BCPAP cells. (**B**). Apoptosis rate from flow cytometry. The apoptosis rate is the number of apoptotic cells divided by the number of total cells × 100. Data are presented as the mean ± SD based on more than three independent experiments (* *p* < 0.05).

Changes in the cell cycle after LINC00887 knockdown were evaluated by flow cytometric analysis at different times depending on the cell line. In the case of TPC1 cells, the LINC00887 mutant showed an increase in the G2-phase population at 72 h relative to control cells. On the other hand, the BCPAP LINC00887 mutant showed a significant increase in the S-phase and G2-phase populations, and a decreased G1-phase population with significant differences at 168 h compared to control cells. In both cases, LINC00887 mutant cells show a delay in the cell cycle by arresting PTC cells in the G2 phase (Figure 6). Therefore, downregulation of LINC00887 may regulate PTC progression by altering the distribution of cell cycle phase. Apoptosis analysis showed that LINC00887 knockdown caused an increased in the percentage of apoptotic BCPAP and TPC-1 cells (*p* < 0.05 Figure 7).

### 2.6. LINC00887 Promotes PD-L1 Downregulation

We performed a western blot assay to analyze the effect of LINC00887 downregulation on the PD-L1 protein. As shown in Supplementary Figure S1, the level of PD-L1 protein was found to be reduced in both the TPC1 and BCPAP LINC00887 mutated cell lines compared to control cells.

### 2.7. In Silico Prediction Secondary Structure of LINC00887 Mutants Differed Significantly from Control Cells

**Figure 8:**
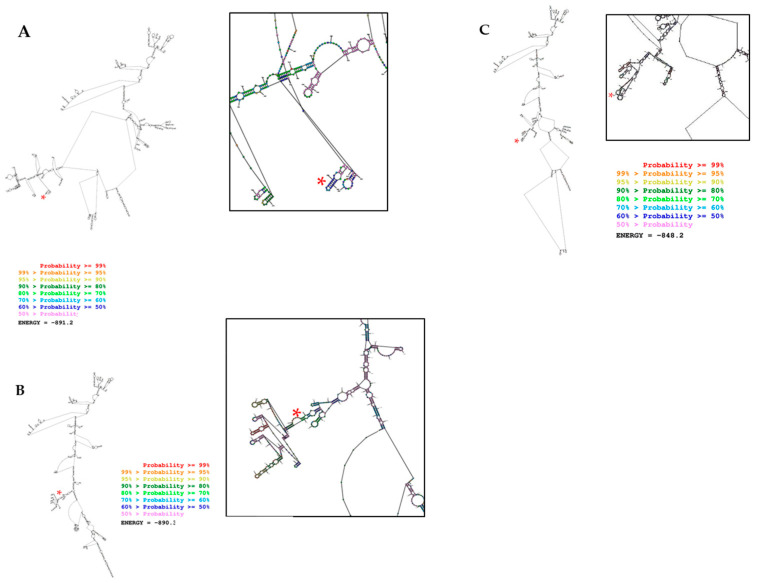
Secondary structure for LINC00887 and base pair probabilities for wild type (**A**), BCPAP LINC00887 mutant (**B**) and TPC1 LINC00887 mutant (**C**). The nucleotide location of the insertion or deletion is indicated by a red asterisk. The red asterisks indicate the nucleotide where the insertion or deletion occurs. Energy is expressed in kcal/mol.

The “Predict a Secondary Structure” web server showed that TPC1 and BCPAP LINC00887 mutant cells altered the RNA secondary structure of LINC00887 compared to the wild type. This program, predicted by free energy minimization (MFE), predicted a local change in the structure around the altered nucleotide, resulting in the deletion of a T in the case of BCPAP LINC00887 mutant cells or the 95 bp deletion in the case of TPC1 LINC00887 mutant cells. Therefore, both changes were predicted to alter the structure–function relationships of LINC00887, leading to a decrease in cell growth (Figure 8).

## 3. Discussion

An increasing number of functional studies have shown that lncRNAs are involved in carcinogenesis and progression of various tumors and may act as oncogenes or tumor suppressors [29]. The altered levels of lncRNAs are associated with the stage and severity of disease, suggesting their potential use in the diagnosis, therapy and prognosis [30]. Increasingly, studies in cancer have described the upregulating or downregulating of certain lncRNAs can regulate cancer progression [31]. Furthermore, it has been described that the expression levels of numerous lncRNAs are dysregulated in the initiation and development of PTC [32]. In particular, several mechanisms have been described by which the lncRNAs could act on PTC [33], including the induction of EMT [15] and the regulation of the Wnt/β-catenin signaling pathway [17], among others. Therefore, it is relatively common that the alteration of PTC-associated lncRNA (e.g., LINC00163, HOTAIR and LINC01638) directly affects proliferation, colony formation, migration and invasion potential [15,16,17,18]. Additionally, these lncRNAs may simultaneously be potential biomarkers for PTC patients and associated with disease prognosis [17,18].

In a previously reported study of our group, we performed a lncRNA array to discover novel lncRNAs associated with PTC [3]. This assay showed that LINC00887 expression was upregulated in PTC samples. Although this lncRNA has been previously described to be involved in several types of cancer [20,21,22,23,24,25,26], its role in PTC has not been fully elucidated. In the current study, we confirmed that LINC00887 expression was upregulated in PTC cell lines (TPC1 and BCPAP). We also performed a loss-of-function approach using CRISPR/Cas9 in these cell lines to investigate the role of LINC00887 in PTC progression. These functional assays showed that LINC00887 knockdown (in both TPC1 and BCPAP LINC00887 mutant cells) resulted in reduced cell growth, cell invasion, colony forming ability, increased apoptosis and a delay in cell cycle progression. These results are consistent with previous studies in which *LINC00887* was also upregulated and its knockdown suppressed cell proliferation, migration and invasion in several tumors, including renal cell carcinoma [20,21,22], nasopharyngeal carcinoma [23], glioma [24], lung carcinoma [25] and tongue squamous carcinoma [26]. In contrast, LINC00887 was downregulated in cervical cancer and its overexpression suppressed the proliferation and invasion[27].

Different mechanisms of action have been described for LINC00887, e.g., in renal cell carcinoma (RCC), Xiong, J., et al. proposed that HEIRCC (LINC00887) regulated RCC cell progression via the epithelial-mesenchymal transition (EMT) [22]. On the other hand, LINC00887 promoted lung carcinoma progression and metastasis by sponging miR-206 to regulate NRP1 expression [25]. In addition, LINC00887, which sponges miR-454-3p, inhibited the cervical cancer progression by activating the FRMD6-Hippo signaling pathway [27]. LINC00887 may also drive the malignant progression of glioma via upregulating Cyclin D1 (CCND1)[24]. At present, the effect of LINC00887 in thyroid cancer is not well understood. Reduced expression of lncRNA in two thyroid cancer cell lines results in decreased expression of PD-L1 protein, the overexpression of which in thyroid cancer patients is associated with poor survival [34].

There is controversy in the literature about the relationship in PTC between PD-L1 expression and the BRAF^V600E^ mutation, which is present in the BCPAP cell line and not in TPC1. Siraj et al. found a significant positive association between PD-L1 expression and the presence of the BRAF^V600E^ mutation, which has been implicated in the pathogenesis and aggressiveness of PTC [35]. Even a study in colorectal cancer (CRC) showed that BRAF^V600E^ can transcriptionally increase PD-L1 expression and enhance apoptosis, suggesting an intrinsic non-immune function of PD-L1 [36]. However, PD-L1 expression has been variably correlated with the molecular profile of thyroid tumors. Whereas some studies have found a significant association with BRAF^V600E^ [37], there are conflicting results [38,39,40,41].

In this study, BCPAP has a higher expression of PD-L1 than TPC1, however, downregulation of LINC00887 leads to decreased protein expression in both cell lines independently of BRAF mutation, suggesting that the etiopatogenic mechanism of LINC00887 in PTC may involve this pathway.

Taking all this into account, the potential oncogenic role of LINC00887 is strengthened. Therefore, we can conclude that LINC00887 promotes PTC cell proliferation, colony formation and cell invasion in vitro, and causes a significant increase in apoptosis and cell cycle delay. On the other hand, the RNA secondary structure prediction of the mutant LINC00887 differed from that of the wild-type, suggesting an alteration in interactions with potential targets. Taken together, these findings suggest that LINC00887 might be a tumor oncogene in PTC.

In summary, LINC00887 is involved in critical processes that are important for the initiation and progression of cancer. These pathways are therefore candidates for activation in PTC. To date, this is the first functional study analyzing the role of LINC00887 in PTC development and suggests that the lncRNA LINC00887 acts as a tumor promoter in PTC. However, further studies are needed to complete this study, for example, RNA-Protein pull-down assay and RNA Immunoprecipitation [42] assay could be performed to elucidate the lncRNA LINC00887-Protein Interactions and to validate the association, which could explain the underlying mechanism of LINC00887.

## 4. Materials and Methods

### 4.1. Cell Culture

Two human papillary thyroid cancer cell lines (TPC1 and BCPAP) were used. The TPC1 cell line was a gift from Dr Velázquez Henar (Autonomous University of Barcelona, Spain) and BCPAP cell line was obtained from Deutsche Sammlung von Mikroorganismen und Zellkulturen, (German) (DSMZ Cat# ACC-273 (established from a metastasizing papillary thyroid carcinoma)). TPC1 and BCAP were cultured in RPMI 1640 w/Glutamax I (Gibco, Thermo Fisher Scientific, Waltham, MA, USA) and Dulbecco’s modified Eagle’s medium (DMEM) culture medium (Gibco, Thermo Fisher Scientific), containing 10% fetal bovine serum (Gibco, Thermo Fisher Scientific), 1% L-glutamine 200 mM (Gibco, Thermo Fisher Scientific), 1% Penicillin-Streptomycin 10,000 U/mL (Gibco, Thermo Fisher Scientific) and 1% Amphotericin B 250 μg/mL (Gibco, Thermo Fisher Scientific) in a 37 °C incubator with 5% CO_2_.

### 4.2. CRISPR/Cas9 Genomic Editing

To study the effect of LINC00887, we designed a CRISPR/Cas9 assay to generate a knockdown model of this gene. We selected exon 2 of LINC00887 as the target of the edition. To disrupt this exon, two single guide RNAs (sgRNAs)-LINC00887 (5′-ACCTGTTTATCGGAGTTGAC-3′ in “+” strand and 5′-TTCTTGGAAACATTCGCAAG-3′ in “-” strand) were designed using the tool “Custom Alt-R CRISPR-Cas9 guide RNA” from Integrated DNA Technologies (IDT, San Diego, CA, USA https://eu.idtdna.com/site/order/designtool/index/CRISPR_CUSTOM; accessed on 20 September 2022) containing fluorescently labelled tracrRNA (Alt-R CRISPR-Cas9 tracrRNA ATTO 550, IDT) and ligated with the Alt-R S.p. Cas9 Nuclease V3 (IDT), to form the ribonucleoprotein (RNP) complex. One million cells were nucleofected with the RNP complex using the Nucleofector System (LONZA, Basel, Switzerland) according to the Amaxa Cell Line Nucleofector Kit V protocol. Cells were then cultured in 6-well plates and incubated at 37 °C for 24 h. Cells were harvested by trypsinization 24 h after nucleofection, resuspended in PBS 1X with 4′,6-diamidino-2-phenylindole, dihydrochloride (DAPI, Thermo ScientificWaltham, MA, USA ) and sorted using MoFlo Astrios Cell Sorter (Beckman Coulter, Barcelona, Spain) to select and collect ATTO positive and DAPI negative cells. Individual clones were picked, grown in complete medium for several weeks and sorted for low, very low or extremely low growth relative to the control in order to amplify them for functional studies and genotyped with Sanger sequencing. This approach was performed together with their respective controls.

### 4.3. Genotyping and Isoform Detection by Sanger Sequencing

Isolation of the genomic DNA in TPC1 and BCPAP candidate cells was performed using the Wizard Genomic DNA Purification Kit (#A1120, Promega, Madrid, Spain) according to the manufacturer’s instructions. Primer pairs were designed using the PrimerQuest Tool (IDT, https://eu.idtdna.com/PrimerQuest/Home/Index, accessed on 20 September 2022), and were used both to detect the isoform expressed in TPC1 and BCPAP cell lines and to analyze the target region of LINC00887 for the designed CRISPR guide, thus determining the sequence changes induced by CRISPR/Cas9 (Supplementary Table S1). PCR products were purified using Illustra ExoProStar 1-Step (GE Healthcare Life Science, Madrid, Spain). The purified PCR product was sequenced by using the BigDye 3.1 Terminator sequencing kit (Applied Biosystems, Waltham, MA, USA) and the ABI 3730xL automated capillary electrophoresis system (Applied Biosystems), according to the manufacturer’s protocol. Sequences were visualized and analyzed using SeqMan NGen version 17.2 (DNASTAR. Madison, WI, USA).

### 4.4. RNA Extraction and Quantitative Real-Time PCR (qRT-PCR)

For the evaluation of LINC00887 expression, total RNA was extracted from TPC1 and BCPAP cells with ReliaPrep RNA Miniprep Systems (Promega, Madison, WI, USA). RNA (1 μg) was reverse transcribed into cDNA using the Prime Script Reverse Transcription System (Takara, Shiga, Japan) according to the manufacturer’s protocol. qRT-PCR reactions were performed using iTaq Universal SYBR Green Supermix (Bio-Rad, Madrid, Spain) in the 7500 Fast Real-Time PCR System (Applied Biosystems) according to the manufacturer’s instructions. The relative expression levels of LINC00887 were calculated by the 2^−ΔΔCT^ method [43] using GAPDH as the internal reference control for the normalization of gene expression statistics. The primer sequences in this assay are listed as follows: LINC00887 forward 5′-CTGCGAAGGAATGACAAAGAAC-3′ and reverse 5′-CTTCGACTGAGGCATCGTT–3′; GAPDH forward 5′-ACATCATCCCTGCCTCTACG-3′ and reverse 5′-CCTGCTTCACCACCTTCTTG-3′.

In addition, the UALCAN online tool (https://ualcan.path.uab.edu/index.html; accessed on 15 October 2023), which contains the Cancer Genome Atlas (TCGA) expression data [44], was used to analyze the differences in the expression profile between normal and tumor tissues and those histological subtypes of thyroid cancer.

### 4.5. Cell Proliferation Assay

Cell Counting Kit-8 (CCK-8, Dojindo, Shanghai, China) was used for cell proliferation assay following the manufacturer’s protocol. According to the growth characteristics of each cell type, TPC1 and BCPAP cells were seeded at a density of 1 × 10^3^ cells/well and 5 × 10^3^ cells/well respectively, and cultured in triplicate in 96-well culture plates. CCK-8 was added to each well and incubated at 37 °C for 3–4 h. Absorbance was measured at a wavelength of 450 nm in the Spectrophotometer Multiskan GO SkyHigh Plate Reader (Thermo Scientific, Waltham, MA, USA).

### 4.6. Colony Formation Assay

A plate colony formation assay was used to measure the rate of cell colony formation. According to the growth characteristics of each cell type, TPC1 were seeded uniformly on 10 mm plates at a density of 100 cells, however, BCPAP were seeded uniformly on 6-well plates at a density of 1000 cells per well. Cells were incubated at 37 °C for one or two weeks, fixed with 4% paraformaldehyde and stained with crystal violet. The number of colonies in each well was counted. Photographs were taken for observation under an inverted light microscope (Olympus Corporation, Madrid, Spain).

### 4.7. Wound Healing Assay

A wound healing assay was performed to assess invasion ability. TPC1 and BCPAP cells (2 × 10^5^ cells/well) were seeded uniformly on a 6-well plate and cultured at 37 °C to 80–90% confluence. The wound was created by scratching the cell layer with a sterile 1000 µL pipette tip. The stripped cells were cultured in serum-free RPMI and DMEM culture medium at 37 °C for 48 h. An inverted light microscope (Olympus Corporation) was used to monitor wound closure at 0, 24 h and 48 h at ×4 magnification. The gap distance was quantitatively evaluated using ImageJ software (version 1.53; National Institutes of Health, Bethesda, MD, USA) and the macro named “Wound_healing_size_tool_updated.ijm”. The migration rate was calculated using the following formula [45]: Wound closure (%) = (distance of initial scratch − distance of closed scratch)/distance of initial scratch.

### 4.8. Cell Cycle Assay

The cell cycle of TPC1 and BCPAP cells was assessed using propidium iodide (Invitrogen, Thermo Fisher) according to the manufacturer’s protocol. TPC1 and BCPAP cells (5 × 10^5^ cells/well) were grown in duplicate on 6-well plates at 37 °C in the complete medium at 80–90% confluence. The medium was changed to medium supplemented with 0.5% FBS for 24 h. After serum starvation, cells were harvested (T0), whereas the rest was maintained by complete medium (10% FBS) and harvested at 72 h in the case of TPC1 cells and at 168 h for BCPAP, according to their specific growth rates. Cells were fixed in 70% ethanol at 4 °C overnight and incubated with RNase A (Roche, Merck, Darmstadt, Germany ) and propidium iodide (Invitrogen, Thermo Fisher) at 37 °C for 30 min in the dark. Both cell cycles were assessed using a BD FACS Canto II flow cytometer (Becton Dickinson, Madrid, Spain) and analyzed using BD FACS Diva Software v8.0.

### 4.9. Apoptosis Assay

To investigate the influence of *LINC00887* in cell apoptosis, TPC-1 and BCPAP cells were analyzed using an Annexin V-FITC Apoptosis Detection kit (BD Biosciences), according to the manufacturer’s instructions. Briefly, cells were harvested and transferred to a 5 mL tube before the addition of 2.5 µL Annexin V and 5 µL propidium iodide (PI). After 30 min incubation in the dark at room temperature, 200 µL of binding buffer was added. Apoptosis was detected with a BD FACS Canto II flow cytometer (Becton Dickinson). Data were analyzed using the BD FACS Diva Software v 8.0.

### 4.10. Western Blotting

For protein extracts, cells were seeded at a density of 250.000 cells/well and harvested after 24 h using RIPA buffer (Sigma-Aldrich, Madrid, Spain). Protein quality was quantified by the Bradford method (ThermoFisher Scientific, Waltham, MA, USA). Protein extracts were separated by 12% SDS-PAGE, an equal amount of protein was transferred onto polyvinylidenefluoride (BioRad, Hercules, CA, USA) membranes, blocked with 5% skimmed milk and incubated with primary antibodies anti-PD-L1 (ab205921) at 4 °C overnight. After incubation with secondary antibodies (anti-rabbit IgG, HRP Linked whole Ab, (Cytiva NA934, Marlborough, MA, USA), Merck, Darmstadt, Alemania, 1:20,000), the bands were visualized by ECL (BioRad) and quantified using ImageLab software 6.0.1 on a ChemiDoc XRS + (BioRad) system. β-Actin was used as an internal control (β-Actin (8H10D10) Mouse mAb Cat# 3700).

### 4.11. In Silico Prediction of the Secondary Structure

To assess the possible effect of the sequence changes produced by CRISPR/Cas9 on the secondary structure of LINC00887, we used the “Predict a Secondary Structure” Web Server version 6.0.1 (https://rna.urmc.rochester.edu/RNAstructureWeb/Servers/Predict1/Predict1.html accessed on 12 December 2022). This server combines several prediction and analysis algorithms to predict a minimum free energy (MFE) structure and find structures with the maximum expected accuracy.

### 4.12. Statistical Analysis

Data resulting from all of the experiments (qRT-PCR, cell proliferation, colony formation, cell invasion, apoptosis and cell cycle assay) were presented as the mean ± SD of at least three independent experiments. Comparisons between the results of the two groups (control versus mutant) were performed with a Student’s *t*-test. A *p* value < 0.05 was considered statistically significant.

## Data Availability

The data that support the findings of this study are available from the corresponding authors upon reasonable request.

## Supplementary Materials

The supporting information can be downloaded at: https://www.mdpi.com/article/10.3390/ijms25031587/s1.

## References

[1] Bogovic Crncic T., Ilic Tomas M., Girotto N., Grbac Ivankovic S. (2020). Risk Factors for Thyroid Cancer: What Do We Know So Far?. Acta Clin. Croat..

[2] Siegel R.L., Miller K.D., Jemal A. (2017). Cancer Statistics, 2017. CA Cancer J. Clin..

[3] Luzon-Toro B., Fernandez R.M., Martos-Martinez J.M., Rubio-Manzanares-Dorado M., Antinolo G., Borrego S. (2019). LncRNA LUCAT1 as a novel prognostic biomarker for patients with papillary thyroid cancer. Sci. Rep..

[4] Du Z., Wang B., Tan F., Wu Y., Chen J., Zhao F., Liu M., Zhou G., Yuan C. (2023). The regulatory role of LncRNA HCG18 in various cancers. J. Mol. Med..

[5] Jendrzejewski J., Thomas A., Liyanarachchi S., Eiterman A., Tomsic J., He H., Radomska H.S., Li W., Nagy R., Sworczak K. (2015). PTCSC3 Is Involved in Papillary Thyroid Carcinoma Development by Modulating S100A4 Gene Expression. J. Clin. Endocrinol. Metab..

[6] Yang J., Ma Y., Gong Y., Gong R., Li Z., Zhu J. (2019). Multiple Simultaneous Rare Distant Metastases as the Initial Presentation of Papillary Thyroid Carcinoma: A Case Report. Front. Endocrinol..

[7] Xing M. (2013). Molecular pathogenesis and mechanisms of thyroid cancer. Nat. Rev. Cancer.

[8] Cai W.Y., Chen X., Chen L.P., Li Q., Du X.J., Zhou Y.Y. (2018). Role of differentially expressed genes and long non-coding RNAs in papillary thyroid carcinoma diagnosis, progression, and prognosis. J. Cell. Biochem..

[9] Sui X., Geng J.H., Li Y.H., Zhu G.Y., Wang W.H. (2018). Calcium channel alpha2delta1 subunit (CACNA2D1) enhances radioresistance in cancer stem-like cells in non-small cell lung cancer cell lines. Cancer Manag. Res..

[10] Huarte M. (2015). The emerging role of lncRNAs in cancer. Nat. Med..

[11] Ge W.J., Huang H., Wang T., Zeng W.H., Guo M., Ren C.R., Fan T.Y., Liu F., Zeng X. (2023). Long non-coding RNAs in hepatocellular carcinoma. Pathol. Res. Pract..

[12] Li C.H., Chen Y. (2013). Targeting long non-coding RNAs in cancers: Progress and prospects. Int. J. Biochem. Cell Biol..

[13] Xing C., Sun S.G., Yue Z.Q., Bai F. (2021). Role of lncRNA LUCAT1 in cancer. Biomed. Pharmacother..

[14] Vecera M., Sana J., Lipina R., Smrcka M., Slaby O. (2018). Long Non-Coding RNAs in Gliomas: From Molecular Pathology to Diagnostic Biomarkers and Therapeutic Targets. Int. J. Mol. Sci..

[15] Gao P., Jiao H., Zhe L., Cui J. (2020). High expression of LINC0163 promotes progression of papillary thyroid cancer by regulating epithelial-mesenchymal transition MITF. Eur. Rev. Med. Pharmacol. Sci..

[16] Di W., Li Q., Shen W., Guo H., Zhao S. (2017). The long non-coding RNA HOTAIR promotes thyroid cancer cell growth, invasion and migration through the miR-1-CCND2 axis. Am. J. Cancer Res..

[17] Wu L., Shi Y., Liu B., Zhao M. (2020). Expression of lncRNA-HOTAIR in the serum of patients with lymph node metastasis of papillary thyroid carcinoma and its impact. Oncol. Lett..

[18] Lv P., Xue Y. (2021). ETS like-1 protein ELK1-induced lncRNA LINC01638 accelerates the progression of papillary thyroid cancer by regulating Axin2 through Wnt/beta-catenin signaling pathway. Bioengineered.

[19] Lin J., Zhang X., Xue C., Zhang H., Shashaty M.G., Gosai S.J., Meyer N., Grazioli A., Hinkle C., Caughey J. (2015). The long noncoding RNA landscape in hypoxic and inflammatory renal epithelial injury. Am. J. Physiol. Renal Physiol..

[20] Wu J., Lin R., Zhang L., Wei Y., Zhang R., Cai W., Hu W. (2022). LINC00887 Fosters Development of Clear Cell Renal Cell Carcinoma via Inhibiting CD8+ T Cell Immune Infiltration. Comput. Math. Methods Med..

[21] Xie J., Zhong Y., Chen R., Li G., Luo Y., Yang J., Sun Z., Liu Y., Liu P., Wang N. (2020). Serum long non-coding RNA LINC00887 as a potential biomarker for diagnosis of renal cell carcinoma. FEBS Open Bio.

[22] Xiong J., Liu Y., Luo S., Jiang L., Zeng Y., Chen Z., Shi X., Lv B., Tang W. (2017). High expression of the long non-coding RNA HEIRCC promotes Renal Cell Carcinoma metastasis by inducing epithelial-mesenchymal transition. Oncotarget.

[23] Yue W.J., Wang Y., Li W.Y., Wang Z.D. (2020). LINC00887 regulates the proliferation of nasopharyngeal carcinoma via targeting miRNA-203b-3p to upregulate NUP205. Eur. Rev. Med. Pharmacol. Sci..

[24] Shen X.M., Han S., Liu N., Xu H.Q., Yan C.X., Yu C.J. (2021). LINC00887 aggravates the malignant progression of glioma via upregulating CCND1. Eur. Rev. Med. Pharmacol. Sci..

[25] Xu L.B., Bo B.X., Xiong J., Ren Y.J., Han D., Wei S.H., Ren X.P. (2021). Long non-coding RNA LINC00887 promotes progression of lung carcinoma by targeting the microRNA-206/NRP1 axis. Oncol. Lett..

[26] Shen T., Xia W., Min S., Yang Z., Cheng L., Wang W., Zhan Q., Shao F., Zhang X., Wang Z. (2021). A pair of long intergenic non-coding RNA LINC00887 variants act antagonistically to control Carbonic Anhydrase IX transcription upon hypoxia in tongue squamous carcinoma progression. BMC Biol..

[27] Li P., Wang J., Zhi L., Cai F. (2021). Linc00887 suppresses tumorigenesis of cervical cancer through regulating the miR-454-3p/FRMD6-Hippo axis. Cancer Cell Int..

[28] Ghafouri-Fard S., Shoorei H., Hussen B.M., Poornajaf Y., Taheri M., Sharifi G. (2022). Interplay between programmed death-ligand 1 and non-coding RNAs. Front. Immunol..

[29] Sultmann H., Diederichs S. (2014). Long noncoding RNA: “LNCs” to cancer. Eur. Urol..

[30] Zhao Y., Wang H., Wu C., Yan M., Wu H., Wang J., Yang X., Shao Q. (2018). Construction and investigation of lncRNA-associated ceRNA regulatory network in papillary thyroid cancer. Oncol. Rep..

[31] Renganathan A., Felley-Bosco E. (2017). Long Noncoding RNAs in Cancer and Therapeutic Potential. Adv. Exp. Med. Biol..

[32] Lan X., Zhang H., Wang Z., Dong W., Sun W., Shao L., Zhang T., Zhang D. (2015). Genome-wide analysis of long noncoding RNA expression profile in papillary thyroid carcinoma. Gene.

[33] Lin R.X., Yang S.L., Jia Y., Wu J.C., Xu Z., Zhang H. (2022). Epigenetic regulation of papillary thyroid carcinoma by long non-coding RNAs. Semin. Cancer Biol..

[34] Wang H., Zhang Z., Yan Z., Ma S. (2020). PD-L1, PDK-1 and p-Akt are correlated in patients with papillary thyroid carcinoma. Adv. Clin. Exp. Med..

[35] Siraj A.K., Parvathareddy S.K., Pratheeshkumar P., Divya S.P., Al-Sobhi S.S., Al-Dayel F., Al-Kuraya K.S. (2021). PD-L1 Is an Independent Prognostic Marker in Middle Eastern PTC and Its Expression Is Upregulated by BRAF^V600E^ Mutation. Cancers.

[36] Feng D., Qin B., Pal K., Sun L., Dutta S., Dong H., Liu X., Mukhopadhyay D., Huang S., Sinicrope F.A. (2019). BRAF^V600E^-induced, tumor intrinsic PD-L1 can regulate chemotherapy-induced apoptosis in human colon cancer cells and in tumor xenografts. Oncogene.

[37] Girolami I., Pantanowitz L., Mete O., Brunelli M., Marletta S., Colato C., Trimboli P., Crescenzi A., Bongiovanni M., Barbareschi M. (2020). Programmed Death-Ligand 1 (PD-L1) Is a Potential Biomarker of Disease-Free Survival in Papillary Thyroid Carcinoma: A Systematic Review and Meta-Analysis of PD-L1 Immunoexpression in Follicular Epithelial Derived Thyroid Carcinoma. Endocr. Pathol..

[38] Ahn S., Kim T.H., Kim S.W., Ki C.S., Jang H.W., Kim J.S., Kim J.H., Choe J.H., Shin J.H., Hahn S.Y. (2017). Comprehensive screening for PD-L1 expression in thyroid cancer. Endocr. Relat. Cancer.

[39] Bastman J.J., Serracino H.S., Zhu Y., Koenig M.R., Mateescu V., Sams S.B., Davies K.D., Raeburn C.D., McIntyre R.C., Haugen B.R. (2016). Tumor-Infiltrating T Cells and the PD-1 Checkpoint Pathway in Advanced Differentiated and Anaplastic Thyroid Cancer. J. Clin. Endocrinol. Metab..

[40] Zwaenepoel K., Jacobs J., De Meulenaere A., Silence K., Smits E., Siozopoulou V., Hauben E., Rolfo C., Rottey S., Pauwels P. (2017). CD70 and PD-L1 in anaplastic thyroid cancer—Promising targets for immunotherapy. Histopathology.

[41] Ahn J., Jin M., Song E., Ryu Y.M., Song D.E., Kim S.Y., Kim T.Y., Kim W.B., Shong Y.K., Jeon M.J. (2021). Immune Profiling of Advanced Thyroid Cancers Using Fluorescent Multiplex Immunohistochemistry. Thyroid.

[42] Chandra Gupta S., Nandan Tripathi Y. (2017). Potential of long non-coding RNAs in cancer patients: From biomarkers to therapeutic targets. Int. J. Cancer.

[43] Liu C., Song Y., Li D., Wang B. (2023). Regulation of the tumor immune microenvironment by the Hippo Pathway: Implications for cancer immunotherapy. Int. Immunopharmacol..

[44] Chandrashekar D.S., Bashel B., Balasubramanya S.A.H., Creighton C.J., Ponce-Rodriguez I., Chakravarthi B., Varambally S. (2017). UALCAN: A Portal for Facilitating Tumor Subgroup Gene Expression and Survival Analyses. Neoplasia.

[45] Zhu Y., Sun W., Jiang X., Bai R., Luo Y., Gao Y., Li S., Huang Z., Gong Y., Xie C. (2023). Differential effects of WRAP53 transcript variants on non-small cell lung cancer cell behaviors. PLoS ONE.

[46] World Medical A. (2013). World Medical Association Declaration of Helsinki: Ethical principles for medical research involving human subjects. JAMA.

