# Supplementary material for: Delving into the Role of lncRNAs in Papillary Thyroid Cancer: Upregulation of LINC00887 Promotes Cell Proliferation, Growth and Invasion
Source: Int J Mol Sci. 2024 Jan 27;25(3):1587. doi: 10.3390/ijms25031587 (PMC10855357; doi:10.3390/ijms25031587)
Supplement: Supplementary file 1 [file ijms-25-01587-s001.zip › ijms-2825624-supplementary-TableS1.pdf]

Supplementary Materials

Supplementary Table S1. Primers sequences used in this study.

	Gene	Forward Primer	Reverse Primer
Genotyping	*LINC00887*_ Ex2	5' – TGGATTCCTCTCCCAT CTT – 3'	5' – GATGGTCCTTCAACTTC TGG – 3'
Isoform detection	*LINC00887*_ Ex3	5' – CTGCGAAGGAATGAC AAAGAAC – 3'	5' – GTCTTAGAGGCAGTGG ATTCTC – 3'
	*LINC00887*_ Ex4	5' – ACGTTGGAGGAAATC AGTAAGT – 3'	5' – CTTGGGCTTCTGGAGCT TTA – 3'
	*LINC00887*_ Ex5	5' – CGTGAGGTTTAGGAG GCTTTAG – 3'	5' – CCTGGGTGTACTTCCTT CTTTG – 3'
	*LINC00887*_ Ex7	5' – TTGCCGGGAATGGGT AAAT – 3'	5' – GCTCAAGTCTCCCTATG AGAAAG – 3'
qRT-PCR	*LINC00887*	5' – CTGCGAAGGAATGAC AAAGAAC – 3'	5' – CTTCGACTGAGGCATC GTT – 3'
	GAPDH	5' – ACATCATCCCTGCCTC TACG – 3'	5' – CCTGCTTCACCACCTTC TTG – 3'
