# Supplementary material for: Delving into the Role of lncRNAs in Papillary Thyroid Cancer: Upregulation of LINC00887 Promotes Cell Proliferation, Growth and Invasion
Source: Int J Mol Sci. 2024 Jan 27;25(3):1587. doi: 10.3390/ijms25031587 (PMC10855357; doi:10.3390/ijms25031587)
Supplement: Supplementary file 1 [file ijms-25-01587-s001.zip › ijms-2825624-supplementary-FigureS1.pdf]

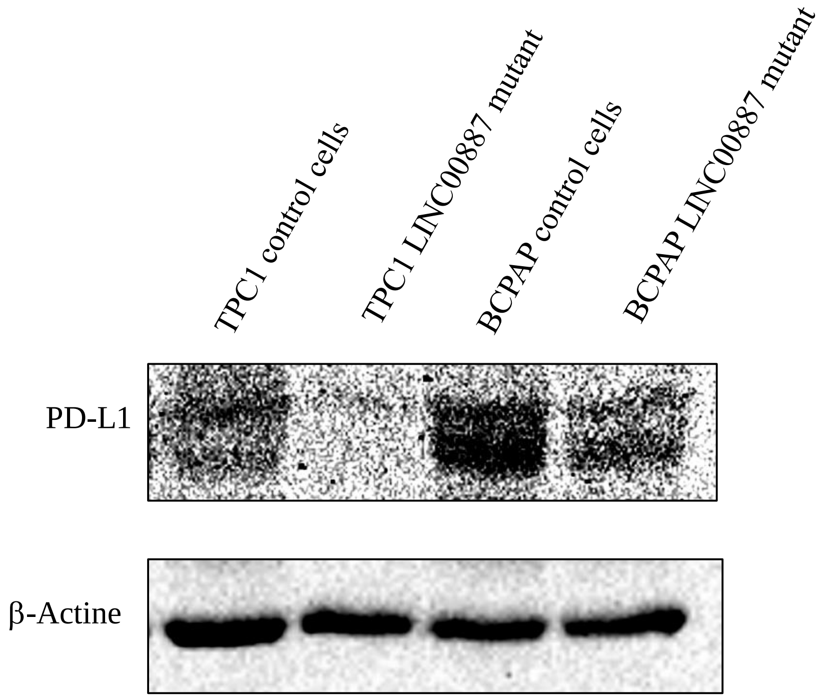

Figure S1. Protein expression of PD-L1 in TPC1 and BCPAP control and LINC0087 mutant cell lines. Western blot assay was used to assess the expression of PD-L1.
